# The care cascade for hepatitis C virus and prognosis of chronic hepatitis C patients treated with antiviral agents in a tertiary hospital

**DOI:** 10.1186/s12876-023-02750-2

**Published:** 2023-04-11

**Authors:** Sung Hwan Yoo, Myung Kim, Sora Kim, Jung Il Lee, Kwan Sik Lee, Hyun Woong Lee, Jin Hong Lim

**Affiliations:** 1 Department of Internal Medicine, Gangnam Severance Hospital, Yonsei University College of Medicine, 211, Eonju-Ro, Gangnam-Gu, Seoul, 06273 Republic of Korea; 2 Department of Surgery, Gangnam Severance Hospital, Yonsei University College of Medicine, 211, Eonju-Ro, Gangnam-Gu, Seoul, 06273 Republic of Korea

**Keywords:** Hepatitis C virus, Sustained virologic response, Liver cirrhosis, Hepatocellular carcinoma

## Abstract

**Background:**

Some studies have analyzed the frequency of HCV RNA testing and actual treatment among anti-HCV positive patients in Korea, which has a low prevalence of HCV infection. This study aimed to analyze the diagnosis process, treatment results, and prognosis according to care cascade in patients who are anti-HCV positive.

**Methods:**

Three thousand two hundred fifty-three anti-HCV positive patients presented to a tertiary hospital between January 2005 and December 2020. The number of patients who underwent HCV RNA testing, treatment, and proportion of sustained virologic response (SVR) according to the type of antivirals was investigated. We investigated the cumulative incidence of hepatocellular carcinoma (HCC) and liver cirrhosis.

**Results:**

Of a total of 3,253 people, 1,177 (36.2%) underwent HCV RNA testing and 858 (72.9%) were positive for HCV RNA. 494 (57.6%) of HCV RNA positive patients received antiviral treatment, and 443 (89.7%) of initiated hepatitis C treatment experienced SVR. Of the 421 treated patients, 16 (14.2%) developed HCC. The cumulative incidence of HCC at 15 years was significantly different according to the presence of liver cirrhosis (10/83, 29.5% vs. 6/338, 10.8%, *p* < 0.001). The cumulative incidences of HCC or liver cirrhosis did not show significant differences according to the presence of SVR_12_ (14/388, 13.2% vs. 2/33, 52.5%, *p* = 0.084, 21/319, 15.0%, vs. 3/22, 28.7%, *p* = 0.051).

**Conclusions:**

Owing to the introduction of direct-acting antivirals, high SVR_12_ was achieved, but the proportion of anti-HCV positive patients who received HCV RNA testing and treatment was not high. HCC surveillance after SVR_12_ is recommended for chronic hepatitis C patients with cirrhosis.

**Supplementary Information:**

The online version contains supplementary material available at 10.1186/s12876-023-02750-2.

## Background

The current prevalence of chronic hepatitis C in Korea is estimated to be approximately 0.6 to 0.8% [1]. Among Korean patients afflicted with hepatitis C, genotype 1b and 2 are the most common types [1]. Overall sustained virologic response rate at 12 weeks (SVR_12_) has improved significantly since the treatment regimen of hepatitis C was switched from interferon drugs to direct-acting antivirals (DAAs), and the efficacy of DAA combination therapy has become more potent. Moreover, DAAs that can treat pangenotypes such as glecaprevir/pibrentasvir (G/P) have been introduced [2]. Nevertheless, patients with chronic hepatitis C often experience DAA treatment failure in the real world. Factors associated with the failure of DAAs include the genetic subtype of chronic hepatitis C, hepatitis C virus (HCV) resistance-associated substitution, liver cirrhosis, a history of previous hepatitis C treatment failure, old age, decreased liver function, and decreased renal function [3]. Furthermore, eradication of HCV by antiviral treatment does not mean that the risk of hepatocellular carcinoma (HCC) is completely eliminated. In particular, patients with advanced fibrosis (F3) or cirrhosis (F4) have a substantial residual risk of HCC, and surveillance is required [4].

It is known that HCC can occur after SVR in the liver of advanced fibrosis (F3). Therefore, early diagnosis and treatment of chronic hepatitis C through a surveillance program is important. Recently, one studied published hepatitis C virus care cascade in one Korea tertiary institution [5]. However, except this study, few studies have analyzed the frequency of HCV ribonucleic acid (RNA) testing among patients who are anti-HCV positive at a tertiary hospital and the proportion of patients who receive HCV treatment in real practice. Therefore, this study aimed to analyze the diagnosis process, treatment results, and prognosis of chronic hepatitis C through a complete investigation of anti-HCV positive patients from January 2005 to December 2020 at a tertiary hospital.

## Methods

### Study population

This retrospective cohort study used data from a single tertiary hospital recorded between January 2005 and December 2020. This study was implemented on people who were positive in the HCV antibody test conducted for various purposes at a tertiary medical institution (Gangnam Severance Hospital). HCV genotyping and serum HCV RNA quantification were performed.

The exclusion criteria were as follows: (1) previous history of HCC or HCC diagnosis within 6 months after HCV treatment, (2) previous history of hepatic decompensation or decompensation development within 6 months after HCV treatment, (3) liver transplant status or transplantation within 6 months after HCV treatment, (4) heavy alcohol consumption (> 30 g/day for males and > 20 g/day for females), (5) toxic hepatitis, and (6) follow-up period less than 6 months.

The study protocol was performed in accordance with the principles of the 1975 Declaration of Helsinki, and approved by the Yonsei University Gangnam Severance Hospital, Institutional Review Board (3–2021-0255). The need for informed consent was waived by the ethics committee/Institutional Review Board of Yonsei University Gangnam Severance Hospital, because of the retrospective nature of the study.

### Baseline workup and treatment plan

The first HCV antibody examination as a baseline study was performed for various reasons. Many patients were found to be anti-HCV positive by accident during preoperative screening test or personal health examinations, and few patients received anti-HCV positive findings in serology tests performed to identify the cause of symptomatic hepatitis. Next, some patients who were anti-HCV positive underwent HCV RNA quantification to confirm HCV infection and HCV genotyping. In some patients who were HCV RNA negative, past HCV infection and false-positive results for anti-HCV were distinguished using the recombinant immunoblot assay (RIBA) test.

Some patients who were confirmed to be HCV RNA positive were started on hepatitis C treatment. HCV treatment is largely divided into interferon-based treatment that stimulates the immune response to HCV and DAA therapy, which directly inhibits the protein components of the HCV. For DAAs, daclatasvir/asunaprevir (DCV/ASV), ledipasvir/sofosbuvir (LED/SOF), sofosbuvir (SOF) and ribavirin, elbasvir/grazoprevir (EBR/GZR), ombitasvir/paritaprevir/ritonavir (OBV/PTV/r), and more recently, the pangenotype agent glecaprevir/pibrentasvir (G/P) were used. The treatment period varied from 8 to 12 weeks, depending on the genotype and presence of cirrhosis. Patients’ HCV RNA was analyzed 4 weeks after the initiation of treatment to confirm patient compliance and evaluate drug according to treatment response, as well as at the end of treatment to evaluate the response at the end of treatment. Moreover, the overall rate of SVR 12 or 24 weeks after completion of therapy (SVR_12_ or SVR_24_) was investigated for all types of HCV treatment. After completion of treatment, each patient underwent abdominal ultrasonography for the surveillance of HCC and fibrosis progression, and the cumulative incidence of HCC and liver cirrhosis was investigated according to SVR_12_ or SVR_24_, baseline liver cirrhosis.

### Laboratory assay

HCV genotyping was performed using the restriction fragment mass polymorphism method.

Serum HCV RNA quantification (lower limit of quantification, 15 IU/mL) was performed with a Cobas 4800 system (Roche Diagnostics GmbH, Mannheim, Germany), which consists of a Cobas × 480 instrument and a Cobas z480 analyzer, to confirm the sustained virological response before and after treatment. In addition, the RIBA test was performed with samples that were HCV RNA negative using MP Diagnostics HCV Blot 3.0 (MP Biomedicals, Singapore).

### Diagnosis of hepatocellular carcinoma and liver cirrhosis

HCC diagnosis was based on the guidelines of the Korean Liver Cancer Association-National Cancer Center [1]. HCC was diagnosed when typical radiologic features such as hypervascularity on arterial phase and washout on portal venous or delayed phase were detected by four-phase multi-detector computed tomography or dynamic contrast-enhanced magnetic resonance imaging. When the diagnosis of HCC was uncertain, it was finally confirmed using two imaging modalities or a liver biopsy [2].

Liver cirrhosis was defined by ultrasonography (US) features such as a blunted, nodular liver surface accompanied by splenomegaly (> 12 cm), in accordance with previous studies [3, 4].

### Statistical analysis

Patients’ baseline characteristics were expressed as mean with standard deviation in the case of continuous variables and numbers with percentages in the case of categorical variables. The construction of the cascade of diagnosis and care is that the number of patients in the previous step corresponds to the denominators at next step. The cumulative incidence rates of HCC and liver cirrhosis were investigated using the Kaplan–Meier method and compared by the log-rank test. The parameters of log-rank test are SVR and liver cirrhosis. Data of patients with HCC or liver cirrhosis at the last follow-up were censored. All missing values were not used for analysis. All statistical analyses were performed using SPSS version 25.0 (IBM Co., Armonk, NY, USA). Statistical significance was set at *P* < 0.05.

## Results

### Baseline characteristics

**Fig. 1:**
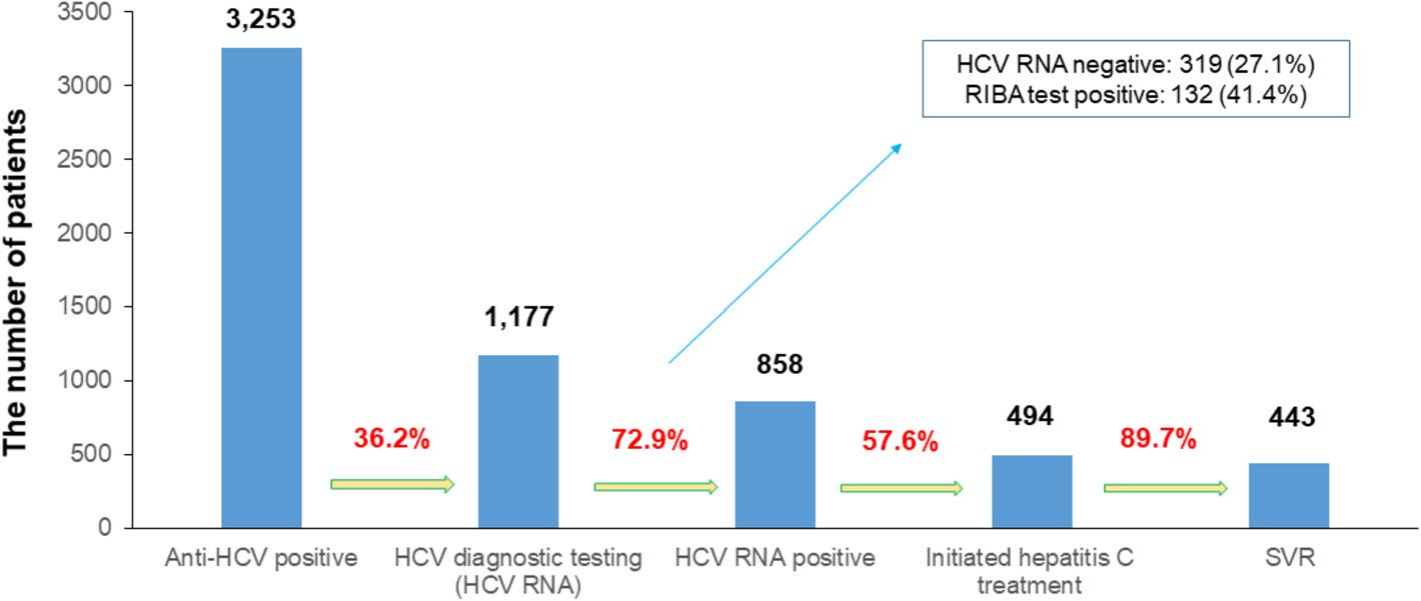
Summary of outcomes of the care cascade in patients who are anti-HCV positive. Of a total of 3,253 people, 1,177 (36.2%) underwent HCV RNA testing and 858 (72.9%) were positive for HCV RNA. A total of 494 (57.6%) patients received antiviral treatment, and 443 (89.7%) experienced SVR

A total of 3,253 patients who were anti-HCV positive and presented to Gangnam Severance Hospital were evaluated between 2005 and 2020. Of these, 1,177 (36.2%) underwent HCV RNA testing for confirmation, with 858 (72.9%) positive for HCV RNA. A total of 319 patients with anti-HCV negative results underwent RIBA testing. A total of 132 (41.4%) tested positive, 181 (56.7%) tested negative, and 86 (1.9%) were indeterminate (Fig. 1).

The baseline characteristics of patients with chronic hepatitis C treated with antiviral treatment (*n* = 494) are shown in Table 1. The proportion of males was 42.5% (*n* = 210), and the proportion of HCV genotype was 58.5% (*n* = 289) for type 1, 38.9% (*n* = 192) for type 2, and 2.4% (*n* = 12) for type 3 (*n* = 12). The mean HCV RNA value was 6.0 log IU/mL, and the mean aspartate aminotransferase and alanine transaminase values were 66.8 and 70.5 IU/L, respectively. The proportions of patients with liver cirrhosis, HCC, diabetes, hypertension, fatty liver, hepatitis B virus and, human immunodeficiency virus coinfection at baseline were 20.9% (*n* = 103), 1.2% (*n* = 6), 23.7% (*n* = 117), 30.2% (*n* = 149), 34.0%(*n* = 168), 3.2% (*n* = 16), and 8.4%(*n* = 41), respectively (Table 1).

**Table 1:** Baseline characteristics of chronic hepatitis C patients treated with antiviral agents

Variables	Antiviral therapy (*n* = 494, %)
Male (%)	210 (42.5)
Genotype	
1	289 (58.5)
2	192 (38.9)
3	12 (2.4)
HCV RNA (log_10_IU/mL)	6.0 ± 0.9
AST (IU/L)	66.8 ± 51.9
ALT (IU/L)	70.5 ± 73.9
Total bilirubin (mg/dL)	0.8 ± 0.5
Albumin (g/dL)	4.2 ± 0.5
Prothrombin time (INR)	1.07 ± 0.19
Platelet (× 10^3^/mm^3^)	191 ± 75
BMI (kg/m^2^)	23 ± 3.5
Liver stiffness (Kpa)	11.8 ± 9.5
Controlled attenuation parameter (dB/m)	226.3 ± 40.0
Antiviral agents	
Pegylated interferon plus ribavirin (%)	212 (42.9)
Daclatasvir/asunaprevir (%)	90 (18.2)
Ledipasvir/sofosbuvir (%)	48 (9.7)
Sofosbuvir and ribavirin	62 (12.6)
Elbasvir/grazoprevir	39 (7.9)
Ombitasvir/paritaprevir/ritonavir	3 (0.6)
Glecaprevir/pibrentasvir	40 (8.1)
Liver cirrhosis (%)	103 (20.9)
HCC (%)	6 (1.2)
Diabetes (%)	117 (23.7)
Hypertension (%)	149 (30.2)
Fatty liver (%)	168 (34.0)
HBV coinfection (%)	16 (3.2)
HIV coinfection (%)	41 (8.4)

Variables are expressed as the mean ± SD or n (%)

*HCV* Hepatitis C virus, *BMI* Body mass index, *HBV* Hepatitis B virus, *HIV* Human immunodeficiency virus, *AST* Aspartate aminotransferase, *ALT* Alanine transaminase, *HCC* Hepatocellular carcinoma

### Progress according to treatment options

Of the 858 HCV RNA positive patients, 494 (57.6%) received first-line antiviral treatment, and of these, 482(95.6%) completed HCV treatments and 443 (89.7%) experienced SVR. Specifically, 212 (42.9%) underwent pegylated interferon plus ribavirin, and 184 (86.8%) experienced SVR_24_. 90 (18.25%) patients who underwent daclatasvir/asunaprevir and 83 (92.2%) patients experienced SVR_12_. Among 48 (9.7%) patients treated with ledipasvir/sofosbuvir, 45 (93.8%) experienced SVR_12_. Among 62 (12.6%) patients who were treated with sofosbuvir and ribavirin, 54 (87.1%) experienced SVR_12_. Among 39 (7.9%) patients treated with elbasvir/grazoprevir, 36 (92.3%) experienced SVR_12_. Among 3 (0.6%) patients treated with ombitasvir/paritaprevir/ritonavir, 3 (100%) experienced SVR_12_. Among 40 (8.1%) patients treated with glecaprevir/pibrentasvir, 38 (95%) experienced SVR_12_ (Supplementary Fig. 1).

### Cumulative incidence of hepatocellular carcinoma and liver cirrhosis

**Fig. 2:**
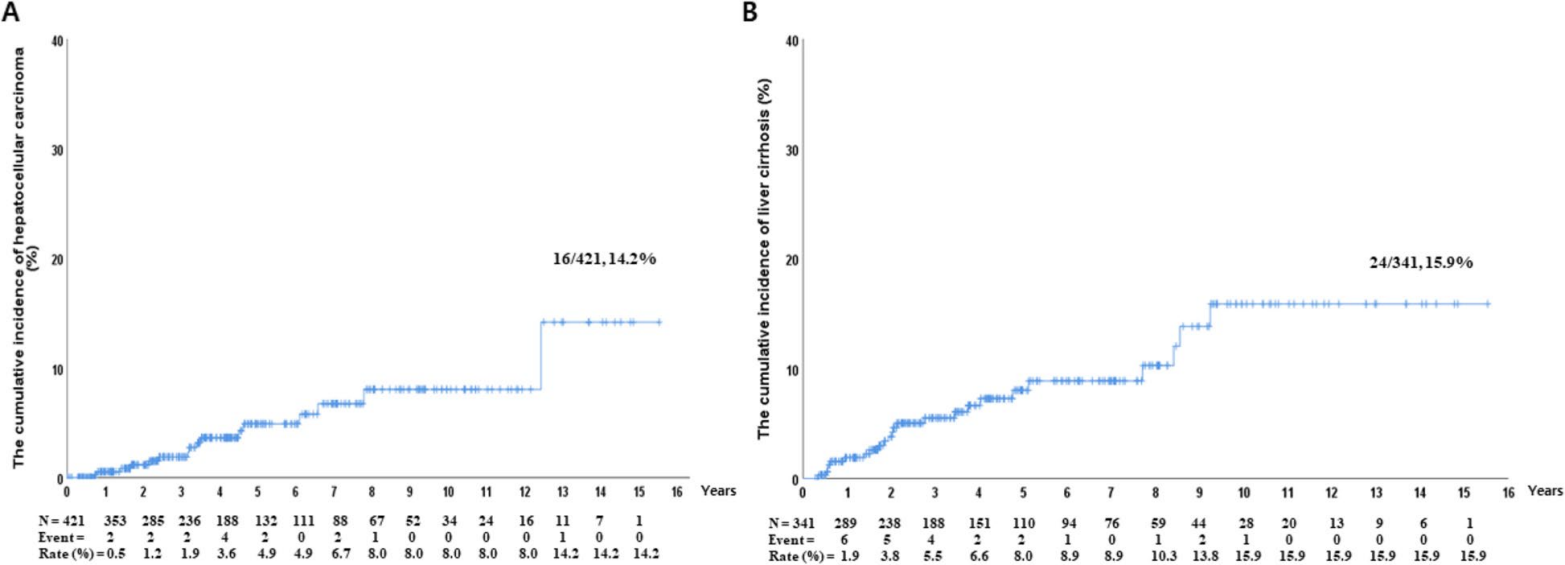
Cumulative incidence rates of HCC (**a**) and liver cirrhosis (**b**). **a** Among 421 patients, 16 developed HCC, and the cumulative incidence of HCC at 15 years was 14.2%. **b** Among 341 patients who had no cirrhosis before treatment, 24 developed liver cirrhosis. The cumulative incidence of liver cirrhosis at 15 years was 15.9%

Among the 421 patients who were followed up for more than 6 months after completion of antiviral therapy, excluding those with HCC at baseline or who developed HCC within 6 months of starting HCV treatment, 16 developed HCC. The cumulative incidence of HCC at 15 years was 14.2%. Of the 16 patients, ten (62.5%) had liver cirrhosis at baseline. A total of 341 patients who had no cirrhosis before treatment were followed up for more than 6 months, and 24 new cases of cirrhosis developed. The cumulative incidence of liver cirrhosis at 15 years was 15.9% (Fig. 2).

**Fig. 3:**
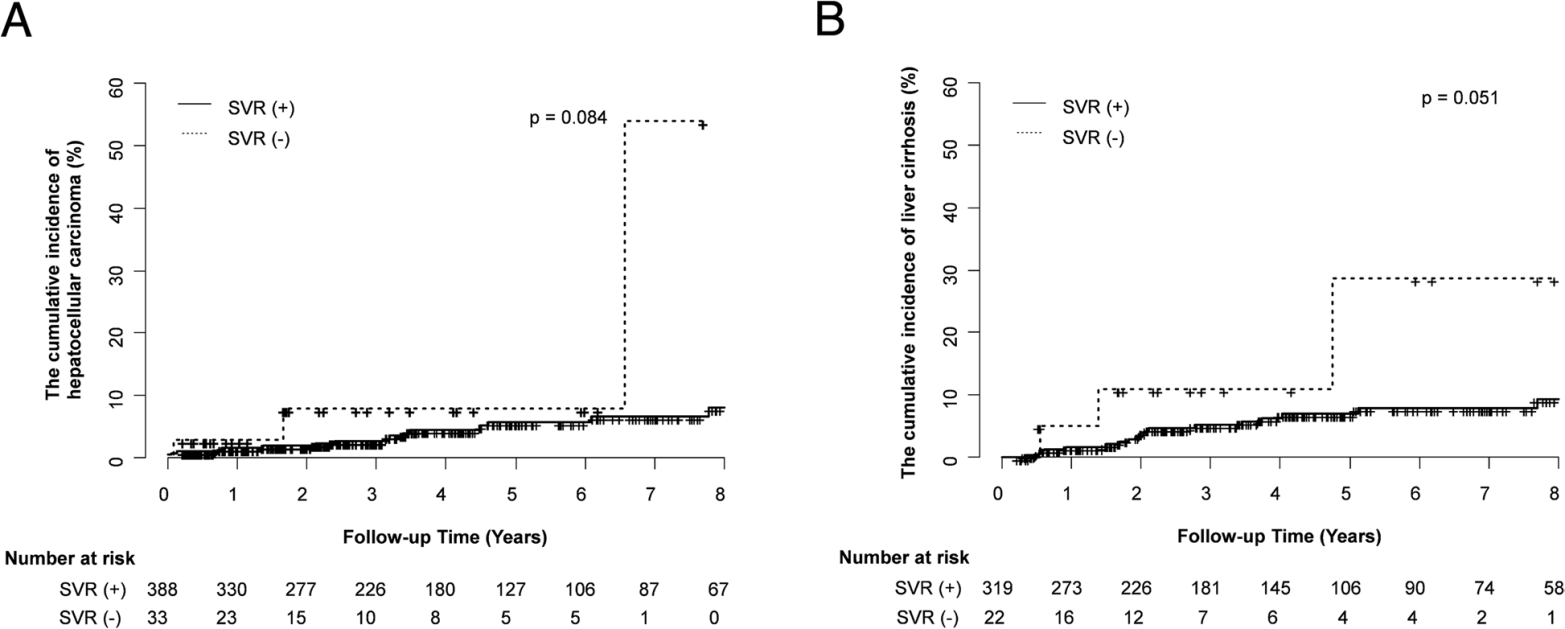
Cumulative incidence rates of HCC (**a**) and liver cirrhosis according to SVR (**b**). **a** The cumulative incidences of HCC did not show statistically significant differences according to the presence of SVR_12_ (14/388, 13.2% vs. 2/33, 52.5%, *p* = 0.084). **b** The cumulative incidences of liver cirrhosis did not show statistically significant differences according to the presence of SVR_12_ (21/319, 15.0%, vs. 3/22, 28.7%, *p* = 0.051)

The cumulative incidence of HCC according to SVR_12_ was analyzed. Among the 388 patients with SVR_12_, 14 developed HCC, with a cumulative incidence rate of 13% at 15 years. In patients without SVR_12_, HCC occurred in two out of 33 patients at 8 years. There was no statistically significant difference in the cumulative incidence rates between the two groups (*p* = 0.084). The cumulative incidence of liver cirrhosis according to SVR_12_ was also investigated. Among the 319 patients with SVR_12_, 21 developed cirrhosis, with a cumulative incidence rate of 15% at 15 years. In patients without SVR_12_, HCC occurred in three out of 22 patients at 8 years. There was no statistically significant difference in the cumulative incidence rate between the two groups (*p* = 0.051) (Fig. 3).

**Fig. 4:**
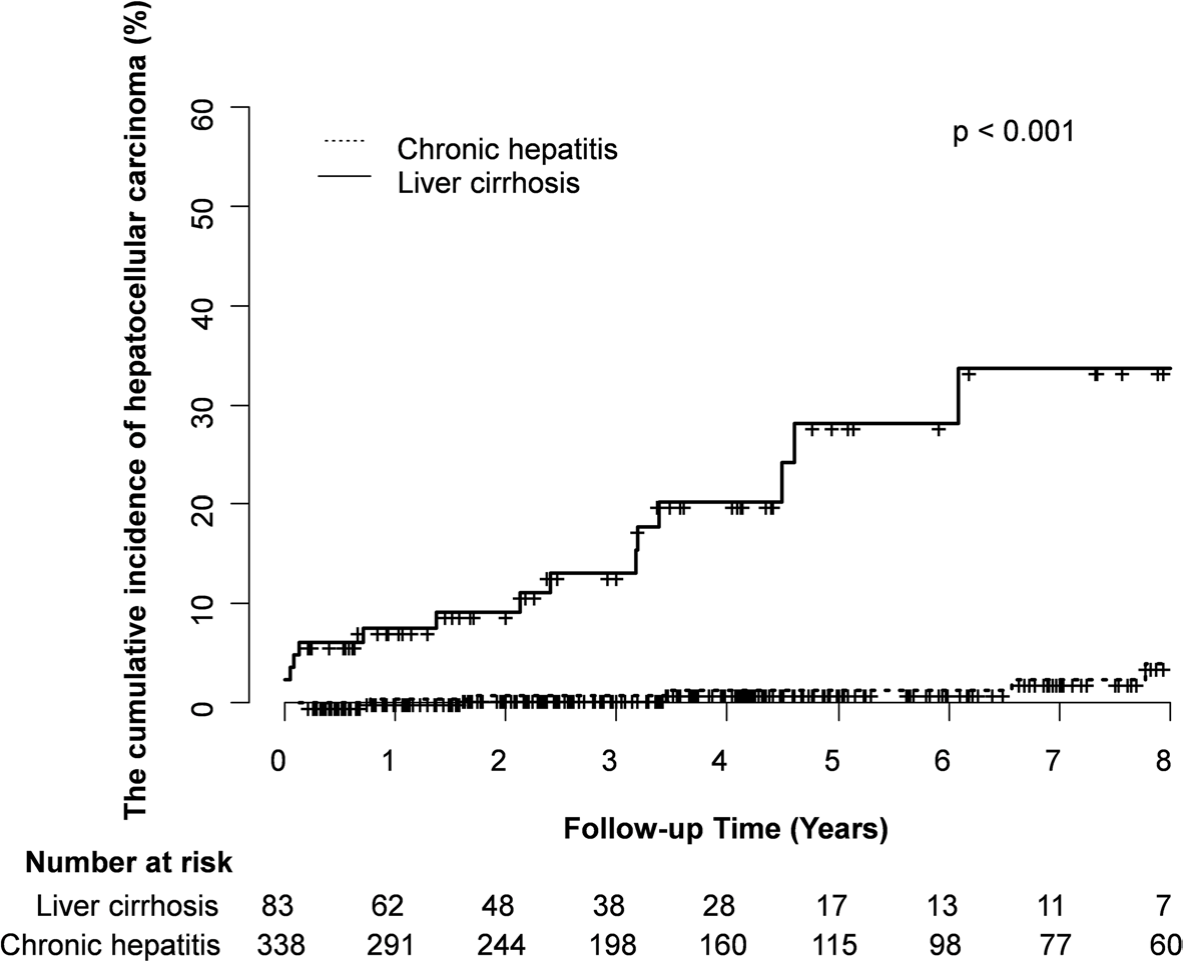
Cumulative incidence rates of HCC according to baseline liver cirrhosis. The cumulative incidence of HCC at 15 years was significantly different according to the presence of liver cirrhosis at the time of diagnosis (10/83, 29.5% vs. 6/338, 10.8%, *p* < 0.001).

The cumulative incidence of HCC according to baseline liver cirrhosis was also analyzed. HCC occurred in six out of 338 patients without cirrhosis, showing a cumulative incidence rate of 10.8% at 15 years, and in ten out of 83 patients with cirrhosis, showing a cumulative incidence rate of 29.5% at 13 years. There was a statistically significant difference in the cumulative incidence between the two groups (*P* < 0.001) (Fig. 4).

## Discussion

The World Health Organization(WHO)’s 2030 global elimination goals for HCV are that 80% of eligible patients are treated, along with a 90% reduction in the incidence of new infections and a 65% reduction in liver-related mortality [5]. However, approximately 400,000 deaths occur annually owing to liver failure and HCC due to chronic HCV infection (currently estimated to be 71 million people worldwide). An estimated 1.75 million people each year are burdened with the disease owing to new HCV infection [6].

With the introduction of DAAs, the goal of hepatitis C elimination has become more attainable [7]. It is paramount to establish an initial screening and treatment program because HCC may occur depending on whether advanced fibrosis (F3) is present at the time of DAA treatment [8]. Efforts in each country to establish screening and treatment for HCV elimination are continuing every year. For example, in April 2015, Georgia, a region with a high prevalence of hepatitis C virus infection, launched the world’s first HCV elimination program to reduce the prevalence of HCV by 90% by 2020 with technical assistance from the Centers for Disease Control and Prevention [9]. Egypt implemented an effective model for HCV screening and treatment delivery in recent years. Between 2014 and 2017, the Egyptian National Committee for Control of Viral Hepatitis provided free DAA-based HCV treatment to more than two million people. As the number of patients with HCV declined in 2018, the Commission introduced a national HCV screening pilot rather than continuing DAAs treatment [10, 11]. On the other hand, in Korea, where the prevalence is low compared to regions with high prevalence, such as Georgia and Egypt, awareness of chronic hepatitis C is low.

Our study had several clinical implications. First, this study assumes significance in that we comprehensively evaluated the number of anti-HCV positive patients who were confirmed and treated at a tertiary medical institution in South Korea. South Korea has not yet implemented a national hepatitis C elimination program. Amongst the anti-HCV positive people included in the current study (3,253), only 1,177 (36.2%) underwent HCV RNA testing for HCV confirmation (Fig. 1). This ratio is considerably lower than that of approximately 67.3% of anti-HCV positive patients in Egypt from October 2018 to September 2019 [5, 12]. Although it is not known exactly why the patient was not tested for HCV RNA, it can be inferred by analyzing the characteristics of the anti-HCV positive patients who were not tested for HCV RNA. There were more cases of preoperative examination than cases of health check-up or symptomatic hepatitis (Supplementary table 1). In the past, it can be assumed that surgery department did not have a proper referral system to the gastrointestinal department, and patients’ awareness of hepatitis C was low. The second reason for less number of people receiving HCV RNA testing is probably due to the lack of understanding of the pathophysiology of chronic hepatitis C and the HCV treatment processes. In most cases, anti-HCV positive results were found incidentally during screening for other purposes such pre-op evaluation or check-up, and not in the acute HCV infection status without any symptom. Patients are likely to be unaware of the need for HCV RNA testing and follow-up for treatment. Among patients with HCV infection confirmed by HCV RNA, the proportion of patients who received treatment was only 57.6%, lower than that in Egypt. This phenomenon might be owing to the cost of chronic hepatitis C treatment itself and a lack of understanding of the hepatitis C treatment process and prognosis.

Second, the cumulative incidence of HCC showed that liver cirrhosis at the time of diagnosis of chronic hepatitis C was more statistically significant than whether SVR_12_ was reached (Figs. 2, 3 and 4). These results are consistent with the results of previous reports [13–17]. It is clear that persistent HCV infection is the strongest factor for HCC in that it induces fibrosis progression and cirrhosis because HCV could induce carcinogenesis indirectly through inflammatory responses or directly via its transcripts or proteins [18, 19]. However, some residual HCC risk could persist after HCV eradication, because HCV-related epigenetic changes and monoclonal micronodules that occur before SVR_12_ are maintained indefinitely even after SVR_12_ [20]. Second, residual fibrosis may progress to advanced fibrosis or cirrhosis owing to other hepatotoxic injury sources (e.g., alcohol, drug, non-alcoholic steatohepatitis) [8]. Therefore, it is necessary to continue HCC surveillance even after reaching SVR_12_ in patients with advanced fibrosis or cirrhosis.

Our study has several limitations. First, this study does not specifically suggest which group to perform the HCV antibody test on as a screening test from a cost-effective point of view. Since the treatment of chronic hepatitis C reduces the risk of HCC and cirrhosis, it is evident that a national screening project to raise people’s awareness should be implemented. However, this study has a drawback in that we did not examine whether screening tests should be performed for all people or only certain groups. Second, this study showed that HCC and liver cirrhosis risk existed even in patients who achieved SVR but did not provide more specific risk stratification for HCC. This is thought to be because our study was conducted over a wide period, from the early days of interferon to the latest DAA treatments, and cirrhosis was only evaluated by abdominal ultrasound. Recently, various tools have been developed to predict HCC risk after SVR, from elastography [17] that can specifically measure the degree of fibrosis to deep learning HCC risk prediction models that use age, sex, race, HCV genotype, and 24 laboratory tests [21]. The HCC surveillance strategy of patients who have reached SVR_12_ requires the introduction of a more specific and easy-to-use model. Third, various clinical characteristics, including their liver function test, the presence of underlying liver disease et al. could not be collected in detail. Fourth, selection bias may occur because only the treated patients were targeted. Fifth, the sample size was small and there may be statistical errors that may occur. For example, the cumulative incidence of HCC and liver cirrhosis does not appear to be statistically related to the presence or absence of SVR in the Fig. 3. This phenomenon is considered to be a statistical error caused by the small number of SVR (-) group with 33 and 22 patients (excluding subjects with liver cirrhosis at baseline), compared to SVR ( +) group. However, the statistical *p* values of Fig. 3A and B were 0.084 and 0.051, respectively, and it was confirmed that there was the trend difference between SVR( +) and SVR(-) group, as p value corresponded to less than 0.1. Clearly, since our study is the retrospective single-institution study, the study population is smaller than other large center multicenter cohort studies, and there are limitations in terms of data availability. However, we are sure that our study could be helpful in establishing guidelines for the current situation in South Korea, which has no public health interventions.

## Conclusions

In conclusion, owing to the introduction of DAA, a high SVR_12_ of chronic hepatitis C was achieved overall, but the proportion of anti-HCV positive patients who received HCV RNA testing and treatment was not high at our tertiary medical institution. This is related to an overall lack of awareness of the purpose and necessity of the HCV screening test and chronic hepatitis C treatment process. Ultimately, it appears that a national screening project should be implemented. Our study shows that by using a public health approach, the care cascade can be greatly improved, and more patients who are anti-HCV positive can be diagnosed and treated with DAAs. For patients with chronic hepatitis C accompanied by baseline cirrhosis, HCC surveillance after SVR_12_ is recommended, and an efficient strategy based on more specific risk stratification is required.

## Supplementary Information

**Additional file 1:** **Supplementary Fig. 1.** Sustained virologic responce according to different HCV treatment types.

**Additional file 2:** **Supplementary table 1.** Characteristics of anti-HCV positive patients not tested for HCV RNA (n=2,076).

## Data Availability

The data used to support the findings of this study are available from the corresponding author upon request.

## Abbreviations

SVR: Sustained virologic response
SVR12: Sustained virologic response rate at 12 weeks
SVR24: Sustained virologic response rate at 24 weeks
HCC: Hepatocellular carcinoma
DAAs: Direct-acting antivirals
G/P: Glecaprevir/pibrentasvir
HCV: Hepatitis C virus
RNA: Ribonucleic acid
RIBA: Recombinant immunoblot assay
DCV/ASV: Daclatasvir/asunaprevir
LED/SOF: Ledipasvir/sofosbuvir
SOF: Sofosbuvir
EBR/GZR: Elbasvir/grazoprevir
OBV/PTV/r: Ombitasvir/paritaprevir/ritonavir
US: Ultrasonography
WHO: World health organization
