# Supplementary figures and images for: The care cascade for hepatitis C virus and prognosis of chronic hepatitis C patients treated with antiviral agents in a tertiary hospital
Source: BMC Gastroenterol. 2023 Apr 11;23:116. doi: 10.1186/s12876-023-02750-2 (PMC10088268; doi:10.1186/s12876-023-02750-2)

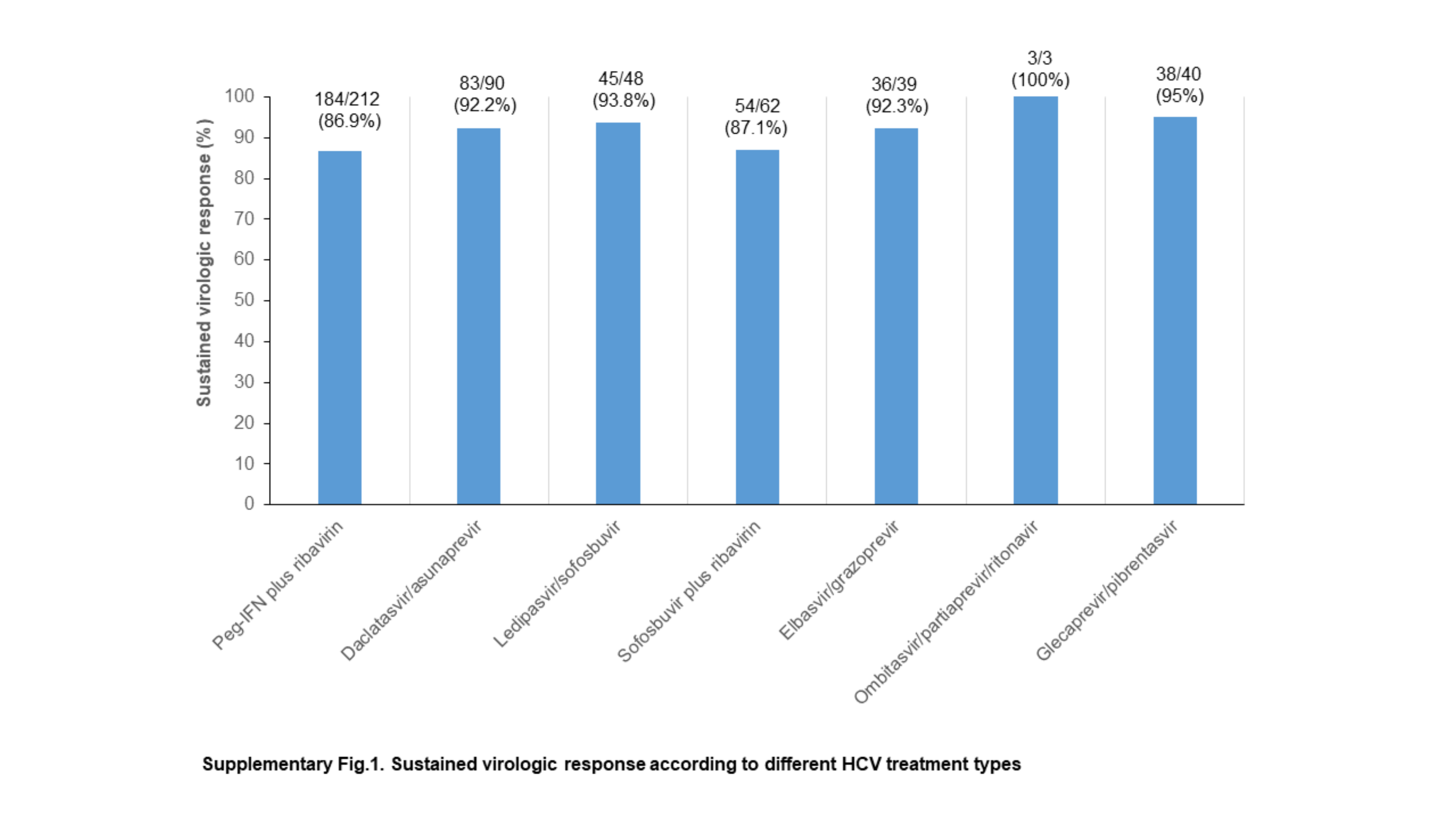

Supplement: Supplementary file 1 — Additional file 1: Supplementary Fig. 1. Sustained virologic responce according to different HCV treatment types. [file 12876_2023_2750_MOESM1_ESM.tif]
