# Supplementary material for: The care cascade for hepatitis C virus and prognosis of chronic hepatitis C patients treated with antiviral agents in a tertiary hospital
Source: BMC Gastroenterol. 2023 Apr 11;23:116. doi: 10.1186/s12876-023-02750-2 (PMC10088268; doi:10.1186/s12876-023-02750-2)
Supplement: Supplementary file 2 — Additional file 2: Supplementary table 1. Characteristics of anti-HCV positive patients not tested for HCV RNA (n=2,076). [file 12876_2023_2750_MOESM2_ESM.docx]

| **Supplementary table 1**. Characteristics of anti-HCV positive patients *not* tested for HCV RNA (n=2,076) | |
| --- | --- |
| Characteristics or comorbidities | Frequency |
| Pre-op evaluation purpose | 1,673 (0.81) |
| Orthopedic surgery | 373 (0.18) |
| Spinal neurosurgery | 263 (0.13) |
| Thyroid cancer surgery | 339 (0.16) |
| Breast cancer surgery | 152 (0.07) |
| General surgery | 132 (0.06) |
| Otolaryngology surgery | 82 (0.04) |
| Eye surgery | 76 (0.04) |
| Thoracic surgery | 61 (0.03) |
| Cardiovascular surgery | 195 (0.09) |
| Health check-up | 395 (0.19) |
| Symptomatic hepatitis patient | 8 (0.01) |
| Variables described as n (%). | |
